# The association between fecal incontinence and asthma among adult Americans: evidence from NHANES 2005–2010

**DOI:** 10.3389/fmed.2025.1564308

**Published:** 2025-06-03

**Authors:** Na Wang, Yongfu Song, Xiaofei Xie, Zhuang Wang, Yongji Wang

**Affiliations:** ^1^College of Traditional Chinese Medicine, Changchun University of Chinese Medicine, Changchun, China; ^2^Department of Pediatrics, The Affiliated Hospital to Changchun University of Chinese Medicine, Changchun, Jilin, China

**Keywords:** asthma, fecal incontinence, cross-sectional study, Americans, NHANES

## Abstract

**Objectives:**

There is a paucity of research exploring the relationship between fecal incontinence (FI) and asthma. This study seeks to evaluate the potential correlation between FI and asthma among adult Americans.

**Methods:**

Utilizing a cross-sectional design, this study comprised a sample of 11,128 adults aged 20 years and older sourced from the National Health and Nutrition Examination Survey (NHANES) conducted between 2005 and 2010. FI is characterized by the involuntary excretion of solid, liquid, or mucus stool occurring at least once a month. Adjusted odds ratios (OR) were calculated using logistic regression models. Subgroup analyses were performed to validate the robustness of the findings.

**Results:**

After adjusting for baseline characteristics, lifestyle habits, and comorbidities, a significant association was observed between FI and an increased risk of asthma (OR: 1.33, 95% CI: 1.1–1.61, *P* = 0.003). Subgroup analysis revealed a significant correlation between FI in females and asthma (OR: 1.36, 95% CI: 1.06–1.73), and this correlation is particularly pronounced in middle-aged and elderly individuals, further supporting the association between FI and asthma.

**Conclusion:**

We found a significant positive correlation between FI and asthma. Females and individuals aged over 45 demonstrate an increased vulnerability to developing asthma. Prompt intervention for individuals experiencing fecal incontinence may mitigate the risk of asthma onset.

## 1 Introduction

Asthma, a prevalent chronic disease affecting both pediatric and adult populations, is characterized by airway inflammation, increased mucus production, and subsequent airflow obstruction (1). In the United States (US), asthma represents a substantial public health burden, with the Centers for Disease Control and Prevention (CDC) reporting a prevalence of approximately 6.8% among the employed adult population (2). This condition imposes a considerable economic cost, estimated at as high as $81 billion annually (3). The repercussions, including exacerbation management, hospital admissions, and ensuing complications, lead to increased healthcare costs and substantially diminish patients’ quality of life (4). Therefore, the precise identification and proactive management of risk factors contributing to asthma’s incidence and prevalence are essential to mitigate this substantial burden.

Fecal incontinence (FI) is typically characterized by the involuntary leakage of solids, liquids, or mucus (5). Epidemiological research reports a prevalence of 8.3% among the non-institutionalized United States population (6, 7). This prevalence underscores the substantial social and economic impact of FI, which significantly impairs patients’ quality of life (8). The mean annual economic burden per affected individual is estimated at $4,110 (9). Although FI and asthma are distinct diseases, they share a common embryonic origin and exhibit analogous structural features in the gastrointestinal and respiratory systems (10, 11), both conditions are also influenced by the parasympathetic nervous system. Nevertheless, the relationship between FI and asthma remains uncertain.

This investigation utilized data from the National Health and Nutrition Examination Survey (NHANES), encompassing three biennial cycles: 2005–2006, 2007–2008, and 2009–2010. Recognized as the most comprehensive nationwide survey in the United States, NHANES encompasses a wide range of demographic characteristics, dietary intake data, and comorbidity profiles. The primary objective of this study was to investigate the association between fecal incontinence and asthma within the adult United States population. Additionally, we assessed the consistency of this association across various subgroups, including age, gender, body mass index (BMI), smoking status, alcohol consumption, poverty income ratio (PIR), and heart disease.

## 2 Materials and methods

### 2.1 Study design and participants

Our investigation into the association between FI and asthma utilized cross-sectional data from the National Health and Nutrition Examination Survey (NHANES) spanning 2005–2010. Administered by the National Center for Health Statistics (NCHS), NHANES is a nationally representative survey designed to assess the health and nutritional status of the United States non-institutionalized population. NHANES adopts a biennial schedule, conducting extensive sampling of approximately 10,000 individuals and incorporating multifaceted health and nutrition evaluations. The study protocol received ethical approval from the NCHS Institutional Review Board (IRB), with all participants providing written informed consent. This ensures ethical compliance. This cross-sectional study adhered to the Strengthening the Reporting of Observational Studies in Epidemiology (STROBE) guidelines (12).

**FIGURE 1:**
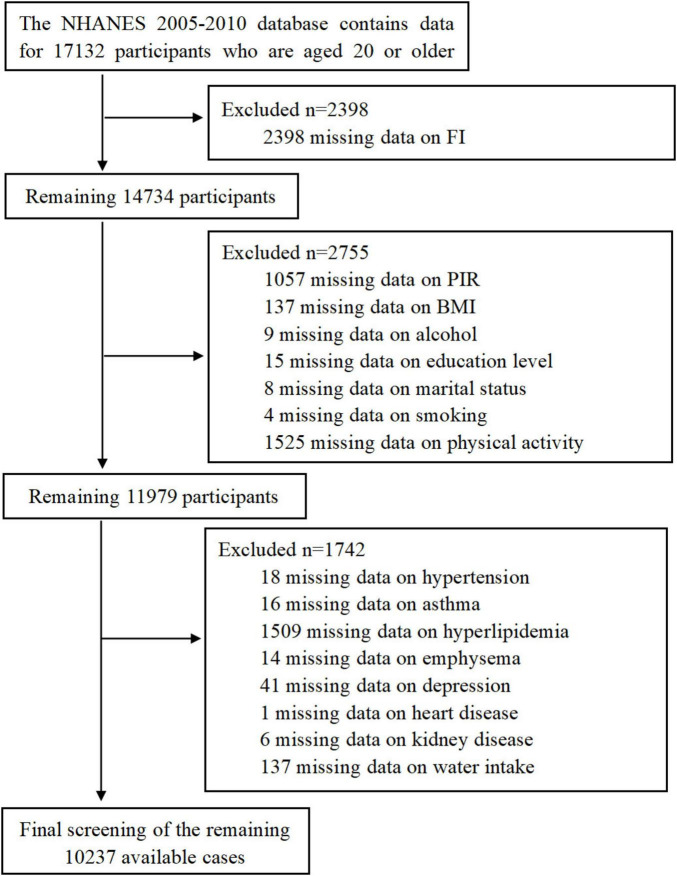
Flow diagram of the sample selection.

This study utilized data from the NHANES database for three consecutive research cycles spanning 2005–2010. These cycles included dedicated questionnaires on gut health. We excluded incomplete data related to gut health and asthma, as well as data entries lacking essential demographic, dietary, and comorbidity information. The detailed workflow is depicted in Figure 1, ultimately resulting in the analysis of 10,237 participants.

### 2.2 FI assessment

The FI severity Index is an important assessment tool in the gastrointestinal health questionnaire. It serves as a metric for assessing the severity of accidental bowel leakage over the past 30 days, encompassing leakage of solid stool, liquid, and mucus (5). The frequency of leakage is categorized based on the number of occurrences: never, 1–3 times per month, once a week, at least twice a week, at least once daily, and at least twice daily. FI is defined as any involuntary leakage of mucus, liquid, or solid stool within the past 30 days, excluding gas leakage. To simplify the analysis process, we classified responses into two categories: no occurrence of bowel leakage (No) and at least one occurrence of bowel leakage (Yes), regardless of the specific frequencies (7).

### 2.3 Asthma assessment

Asthma-related data were collected through the medical questionnaire administered by the NHANES. The survey questions included: (1) Has a doctor or other healthcare professional told you that you have asthma? (2) Do you still have asthma? (3) Have you had an asthma attack in the past year? (4) Have you visited the emergency room or urgent care center due to an asthma attack in the past year? If any of these four questions are answered positively, the respondent is considered to have asthma; otherwise, the respondent is considered not to have asthma.

### 2.4 Covariates

In this study, we analyzed the following covariates: age, gender (male, female), race/ethnicity (Mexican American, other Hispanic, non-Hispanic white, non-Hispanic black, other races), marital status (married/living with partner, widowed/divorced/separated/never married), education level (< high school, high school graduate, > high school), BMI (obesity: BMI ≥ 30 kg/m^2^, overweight: BMI 25–29.9 kg/m^2^, normal weight: BMI < 25 kg/m^2^), and PIR (poor: PIR < 1, not poor: PIR ≥ 1).

Smoking status was categorized into three groups according to the CDC’s standardized criteria for population health surveys: never smokers, former smokers, and current smokers. Never smokers encompass individuals who have never initiated smoking or have smoked fewer than 100 cigarettes prior to the interview. Former smokers are individuals who have smoked a total of 100 cigarettes but have abstained from smoking at the time of the interview. Current smokers are those who have smoked a cumulative total of 100 or more cigarettes in their lifetime and continue to smoke at the time of the interview. Alcohol consumption was categorized into two groups: drinking and non-drinking. Consuming more than 12 drinks per year was defined as drinking, whereas consuming fewer than 12 drinks was considered non-drinking. Physical activity was classified into three levels: inactive, moderate, and vigorous. Dietary data, including fiber intake, energy intake, and total plain water consumption, were collected using two non-consecutive 24 h dietary recalls. The first interview was conducted in person at the mobile examination center, and the second was administered via telephone 3–10 days later, in accordance with the NHANES protocol. This approach minimizes intra-individual variability and enhances the accuracy of dietary estimates compared to single-day assessments. When both days of data were available, the average was calculated; otherwise, the first day’s data were used as the representative value.

The associated complications include diabetes, kidney disease (renal failure, kidney stones), heart disease (heart failure, coronary heart disease, angina, heart attack), hypertension, hyperlipidemia, emphysema, and depression. Participants were asked whether they had been informed by doctors or other healthcare professionals about having diabetes, renal failure, kidney stones, heart failure/coronary heart disease/angina/heart attack, hypertension, hyperlipidemia, or emphysema. Diabetes-related questions also included whether participants used insulin or took antidiabetic medications to lower blood sugar. Depression-related data were collected using the Patient Health Questionnaire-9 (PHQ-9), which comprises nine screening items and generates a total score ranging from 0 to 27. Individuals scoring ≥ 10 are classified as having depression based on established criteria. All of these questions were classified as binary variables with “yes” or “no” answers.

### 2.5 Statistical analyses

Descriptive analysis was performed on all participant data. For continuous variables, mean and standard deviation (SD) or median and interquartile range were used for analysis. Categorical variables were presented as proportions (%). The chi-square test was used to compare categorical variables, whereas the *t*-test was used for continuous variables. Logistic regression models were applied to analyze the relationship between FI and asthma. Both unadjusted and multivariable adjusted models were implemented: Model I without any covariate adjustment; Model II adjusted for gender, age, race/ethnicity, marital status, and education level; Model III further adjusted for smoking status, alcohol consumption, physical activity, BMI, and PIR; Model IV additionally adjusted for dietary fiber intake, energy intake, and water intake; Model V included all covariates from Model IV plus diabetes, heart disease, kidney disease, hyperlipidemia, hypertension, depression, and emphysema. Subgroup analyses were conducted across age, gender, BMI, presence of diabetes and hyperlipidemia, and history of heart disease to evaluate the consistency of the association between FI and asthma. Additionally, a sensitivity analysis was performed to enhance the robustness of the findings. Statistical significance was determined by comparing adjusted ORs and their corresponding 95% confidence intervals (CIs).

The statistical software package R (The R Foundation^1^) and Free Statistics software version1.9.2 were utilized for all analyses. A two-tailed test was employed, with a significance level of *P* < 0.05 indicating statistically significant differences.

## 3 Results

### 3.1 Baseline characteristics

This study collected data from 10,237 participants across three cycles, identifying 952 individuals with FI, corresponding to a prevalence rate of 9.3%. Table 1 summarizes participants’ demographic profiles, lifestyle habits, and comorbidities. Our findings revealed that FI was more prevalent among older individuals, females, non-Hispanic whites, those with marital statuses of married/living with partner, individuals with higher than high school education, those categorized as non-poor, individuals without emphysema or depression, hypertensive individuals, alcohol consumers, obese individuals, and the physically inactive population. Additionally, dietary fiber intake, energy intake, and water consumption were significantly reduced among individuals with FI. Our findings show that the proportion of FI patients was 44.5% among non-smokers and 55.5% among smokers, suggesting that FI is more prevalent in populations with cigarette exposure. Furthermore, the prevalence of asthma was higher (18.7%) among FI individuals than among those without FI (13.1%).

**TABLE 1:** Data characteristics of the participants.

Variables	Total *N* = 10,237	Without FI *N* = 9,285	With FI *N* = 952	*P*-value
**Gender, *n* (%)**				< 0.001
Male	5,011 (48.9%)	4,600 (49.5%)	411 (43.2%)	–
Female	5,226 (51.1%)	4,685 (50.5%)	541 (56.8%)	–
**Age, mean ± SD**	50.6 ± 17.2	49.8 ± 17.2	58.9 ± 15.3	< 0.001
**Total plain water drink, mean ± SD**	16.4 ± 8.7	16.4 ± 8.7	15.8 ± 8.8	< 0.001
**Total energy intake, mean ± SD**	2039.3 ± 846.7	2043.3 ± 849.6	2000.7 ± 817.1	0.139
**Dietary fiber intake, mean ± SD**	703.6 (259.2, 1333.1)	711.0 (261.1, 1362.0)	602.7 (214.8, 1155.4)	0.033
**BMI, *n* (%)**				0.001
Obesity	4,033 (39.4)	3,605 (38.8)	428 (45)	–
Overweight	3,596 (35.1)	3,291 (35.4)	305 (32)	–
Normal weight	2,608 (25.5)	2,389 (25.7)	219 (23)	–
**Race/ethnicity, *n* (%)**				<0.001
Non-Hispanic white	5,396 (52.7)	4,822 (51.9)	574 (60.3)	–
Non-Hispanic black	1,883 (18.4)	1,720 (18.5)	163 (17.1)	–
Mexican American	1,707 (16.7)	1,594 (17.2)	113 (11.9)	–
Other Hispanic	864 (8.4)	798 (8.6)	66 (6.9)	–
Other race	387 (3.8)	351 (3.8)	36 (3.8)	–
**Marital status, n (%)**				<0.001
Married/living with partner	6,498 (63.5)	5,960 (64.2)	538 (56.5)	–
Widowed/divorced/separated/never married	3,739 (36.5)	3,325 (35.8)	414 (43.5)	–
**Education level, *n* (%)**				0.002
< High school	1,045 (10.2)	943 (10.2)	102 (10.7)	–
Completed high school	1,541 (15.1)	1,362 (14.7)	179 (18.8)	–
> High school	7,651 (74.7)	6,980 (75.2)	671 (70.5)	–
**Alcohol, *n* (%)**				0.189
No	2,857 (27.9)	2,574 (27.7)	283 (29.7)	–
Yes	7,380 (72.1)	6,711 (72.3)	669 (70.3)	–
**Smoking, *n* (%)**				<0.001
Never	5,402 (52.8)	4,978 (53.6)	424 (44.5)	–
Former	2,729 (26.7)	2,408 (25.9)	321 (33.7)	–
Current	2,106 (20.6)	1,899 (20.5)	207 (21.7)	–
**PIR, *n* (%)**				0.275
PIR < 1	1,825 (17.8%)	1,643 (17.7%)	182 (19.1%)	–
PIR ≥ 1	8,412 (82.2%)	7,642 (82.3%)	770 (80.9%)	–
**Physical activity, *n* (%)**				< 0.001
Inactive	4,761 (46.5%)	4,271 (46%)	490 (51.5%)	–
Moderate	2,931 (28.6%)	2,643 (28.5%)	288 (30.3%)	–
Vigorous	2,545 (24.9%)	2,371 (25.5%)	174 (18.3%)	–
**Diabetes, *n* (%)**				< 0.001
No	8,955 (87.5%)	8,210 (88.4%)	745 (78.3%)	–
Yes	1,282 (12.5%)	1,075 (11.6%)	207 (21.7%)	–
**Heart disease, *n* (%)**				< 0.001
No	9,347 (91.3%)	8,561 (92.2%)	786 (82.6%)	–
Yes	890 (8.7%)	724 (7.8%)	166 (17.4%)	–
**Kidney disease, *n* (%)**				< 0.001
No	9,225 (90.1%)	8,440 (90.9%)	785 (82.5%)	–
Yes	1,012 (9.9%)	845 (9.1%)	167 (17.5%)	–
**Hyperlipidemia, *n* (%)**				< 0.001
No	6,429 (62.8%)	5,973 (64.3%)	456 (47.9%)	–
Yes	3,808 (37.2%)	3,312 (35.7%)	496 (52.1%)	–
**Hypertension, *n* (%)**				< 0.001
No	1,548 (15.1%)	1,443 (15.5%)	105 (11%)	–
Yes	8,689 (84.9%)	7,842 (84.5%)	847 (89%)	–
**Depression, *n* (%)**				< 0.001
No	9,387 (91.7%)	8,613 (92.8%)	774 (81.3%)	–
Yes	850 (8.3%)	672 (7.2%)	178 (18.7%)	–
**Emphysema, *n* (%)**				< 0.001
No	9,991 (97.6%)	9,084 (97.8%)	907 (95.3%)	–
Yes	246 (2.4%)	201 (2.2%)	45 (4.7%)	–
**Asthma, *n* (%)**				< 0.001
No	8,846 (86.4%)	8,072 (86.9%)	774 (81.3%)	–
Yes	1,391 (13.6%)	1,213 (13.1%)	178 (18.7%)	–

FI, fecal incontinence, BMI, body mass index, PIR, poverty income ratio.

### 3.2 Association between FI and asthma

Univariate logistic regression analysis revealed significant associations between asthma and various factors, including gender, age, race/ethnicity, marital status, education level, PIR, BMI, smoking status, water intake, dietary fiber intake, diabetes, heart disease, kidney disease, emphysema, depression, FI and hypertension (*P* < 0.05) (Table 2). Table 3 presents findings from a multivariate logistic regression analysis examining the relationship between FI and asthma. In Model I, FI was significantly associated with an increased risk of asthma (OR: 1.53, 95% CI: 1.29–1.82, *P* < 0.001). Even after adjusting for gender, age, race/ethnicity, marital status, and education level in Model II, the association between FI and asthma remained statistically significant (OR: 1.59, 95% CI: 1.33–1.90, *P* < 0.001). After further adjusting for smoking status, alcohol consumption, physical activity, BMI, and PIR in Model III, the association between FI and asthma remained statistically significant (OR: 1.5, 95% CI: 1.25–1.8, *P* < 0.001). Even after adjusting for dietary fiber intake, energy intake, and water intake in Model IV, a significant association between FI and asthma remained (OR: 1.51, 95% CI: 1.26–1.81, *P* < 0.001). Finally, in Model V, after adjusting for comorbidities such as diabetes, heart disease, hyperlipidemia, hypertension, depression, emphysema, and renal disorders, a stable association was observed (OR: 1.33, 95% CI: 1.10–1.61, *P* = 0.003).

**TABLE 2:** Univariate regression analysis.

Variable	OR_95 CI	*P*-value
**Gender:** female vs. male	1.36 (1.21–1.53)	< 0.001
**Age**	0.99 (0.99–0.99)	< 0.001
**Race/ethnicity: ref. = Mexican American**		
Other Hispanic	2.18 (1.68–2.83)	< 0.001
Non-Hispanic white	2.21 (1.81–2.69)	< 0.001
Non-Hispanic black	2.29 (1.83– 2.85)	< 0.001
Other race	2.34 (1.68–3.26)	< 0.001
**Marital status: ref. = married/living with partner**		
Widowed/divorced/separated/never married	1.4 (1.25–1.57)	< 0.001
**Education level: ref. = high school**		
Completed high school	1.71 (1.33–2.19)	< 0.001
> High school	1.58 (1.27–1.97)	< 0.001
**PIR:** yes vs. no	0.71 (0.62– 0.81)	< 0.001
**BMI:** ref. = obesity		
Overweight	0.6 (0.53–0.69)	< 0.001
Normal	0.66 (0.57–0.76)	< 0.001
**Physical activities: ref. = inactive**		
Moderate	0.96 (0.84–1.1)	0.524
Active	1.01 (0.88–1.16)	0.928
**Smoking: ref. = never**		
Former	1.19 (1.04–1.37)	0.011
Current	1.57 (1.37–1.81)	< 0.001
**Alcohol:** yes vs. no	1.01 (0.89–1.14)	0.938
Total plain water intake	0.98 (0.98–0.99)	0.031
Total energy intake	1 (1–1)	0.158
Dietary fibre intake	1 (1–1)	< 0.001
**Diabetes:** yes vs. no	1.44 (1.23–1.68)	< 0.001
**Heart disease:** yes vs. no	1.49 (1.25–1.79)	< 0.001
**Kidney disease:** yes vs. no	1.57 (1.32–1.85)	< 0.001
**Hyperlipidemia:** yes vs. no	1.02 (0.87–1.19)	0.85
**Hypertension:** yes vs. no	1.31 (1.17–1.47)	< 0.001
**Emphysema:** yes vs. no	6.42 (4.97–8.3)	< 0.001
**Depression:** yes vs. no	2.25 (1.9–2.66)	< 0.001
**Fecal incontinence:** yes vs. no	1.53 (1.29–1.82)	< 0.001

BMI, body mass index, PIR, poverty income ratio.

**TABLE 3:** Multivariate regression analysis of the association between fecal incontinence and asthma.

Variable	Without FI OR (95% CI)	With FI OR (95% CI)	*P*-value
Model I	1 (ref)	1.53 (1.29–1.82)	< 0.001
Model II	1 (ref)	1.59 (1.33–1.90)	< 0.001
Model III	1 (ref)	1.5 (1.25–1.8)	< 0.001
Model IV	1 (ref)	1.51 (1.26–1.81)	< 0.001
Model V	1 (ref)	1.33 (1.1–1.61)	0.003

Model I: no adjusted. Model II: adjusted for age + gender + race/ethnicity + marital status + education level. Model III: Model II + smoking status + alcohol consumption + physical activity + BMI + PIR. Model IV: Model III + plain water + energy + dietary fiber. Model V: Model IV + diabetes + hypertension + hyperlipidemia + heart disease + kidney disease + depression + emphysema.

**FIGURE 2:**
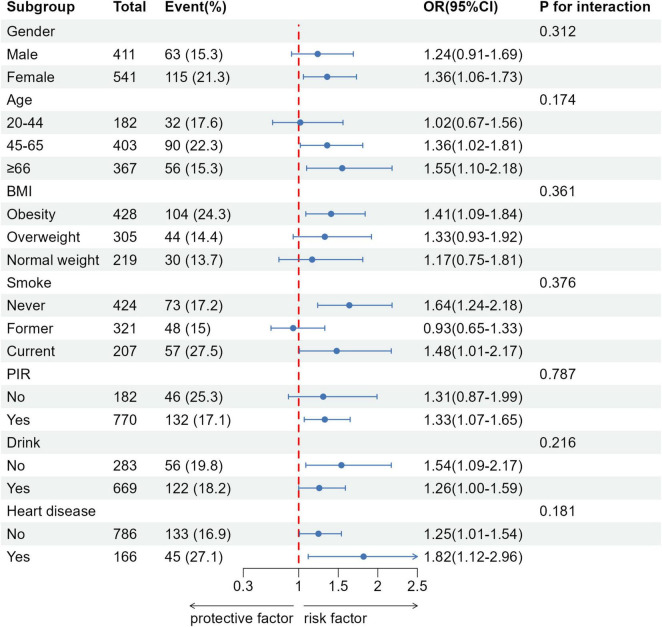
Relationship between fecal incontinence and asthma in different subgroups.

The subgroup analysis results are presented in Figure 2. No significant interactions were observed when examining subgroups based on gender, age, BMI, PIR, alcohol consumption, smoking status, and heart disease (all *P* > 0.05). However, a significant gender disparity was observed in the association between FI and asthma. Among women, there was a significant correlation between FI and asthma (OR: 1.36, 95% CI: 1.06–1.73). Additionally, among participants aged 45–65, a significant association was observed between FI and asthma (OR: 1.36, 95% CI: 1.02–1.81), and this association remained significant among those aged 66 and older (OR: 1.55, 95% CI: 1.10–2.18).

In the sensitivity analysis, we excluded participants with extreme values in dietary fiber intake, energy intake, and total plain water consumption. We examined the relationship between FI and asthma and found that the results remained robust (OR: 1.65, 95% CI: 1.25–2.19, *P* < 0.001) (Supplementary Table).

## 4 Discussion

Our research has made several findings. Firstly, individuals with FI are more susceptible to asthma than those without FI. Secondly, after adjusting for various covariates, a significant correlation between FI and asthma is found. Thirdly, women and age ≥ 45 are identified as factors that significantly increase the likelihood of developing asthma in individuals with FI.

Previous research has established a significant correlation between constipation and asthma (13). However, limited attention has been given to the association between FI and asthma. Although only a few studies have investigated this relationship, an epidemiological survey of elderly individuals in Taiwan revealed a significant correlation between FI and asthma within this population (14). Our research findings indicate a significant positive correlation between FI and asthma, consistent with previous studies. However, our study includes a larger sample size of the American population and incorporates other factors associated with asthma, further strengthening this association. Subgroup analysis reveals that the relationship between FI and asthma is particularly strong among individuals aged 45 and above, consistent with a study conducted in Taiwan (14).

Both FI and asthma are complex conditions arising from a combination of diverse factors, with their pathophysiology potentially involving multiple mechanisms. The cross-sectional design of our study precludes definitive conclusions about causality; however, bidirectional pathways may plausibly exist between these conditions. Impaired epithelial barrier function may underlie both conditions. Research indicates that intestinal and airway epithelial structures share features such as columnar epithelium, goblet cells, and mucus glands due to their common embryological origins, suggesting analogous foundational structures (10, 11). Studies have demonstrated impaired epithelial barrier function in both inflammatory bowel disease and asthma (15, 16). On one hand, FI may contribute to asthma through impaired intestinal epithelial barrier function, allowing microbial translocation or proinflammatory signals to enter systemic circulation, thereby exacerbating airway inflammation (10). Studies have shown that individuals with ulcerative colitis are at a significantly greater risk of developing asthma than healthy controls (17). Conversely, chronic airway inflammation in asthma is linked to systemic immune activation, which may disrupt gut mucosal integrity or neuromuscular function. Asthma-induced chronic and recurrent coughing can result in a significant increase in intra-abdominal pressure, which may stress the pelvic floor structures. This pressure-related mechanism could contribute to FI (18).

Furthermore, altered innate microbial recognition and abnormal inflammatory and immune responses of epithelial cells to environmental factors and pathogens have been identified (16, 19). On the other hand, neural dysfunction may also be a significant factor. The autonomic nervous system plays a crucial role in regulating respiratory and gastrointestinal functions (20), the enteric nervous system, a branch of the autonomic nervous system, extends throughout the digestive tract to regulate gastrointestinal function (21). Additionally, the parasympathetic nerves, innervated by the vagus nerve, control peristalsis in the gastrointestinal tract from the esophagus to the splenic flexure of the colon (22). Parasympathetic nerve dysfunction can affect anal sphincter tone and rectal sensitivity, leading to fecal incontinence (23). In asthma, autonomic nervous system imbalance occurs, particularly increased parasympathetic activity, which releases acetylcholine and causes bronchoconstriction (24). The cell bodies controlling lung and airway sensory neurons are primarily located within the vagus nerve (25).

The microbiota bidirectionally regulates asthma and FI pathogenesis via the gut-lung axis. Microbiota imbalance at any specific site may contribute to the development of diseases in distant organs or tissues (26). Short-chain fatty acids (SCFAs) - primarily produced by gut microbiota fermenting dietary fiber - include acetic acid, propionic acid, and butyric acid. These metabolites modulate immune cell function by activating G protein-coupled receptors. Specifically, SCFAs enhance regulatory T cell development, suppress allergic inflammation, and reduce Th2-type cytokine production, including IL-4, IL-5, and IL-13, in allergic asthma (27). Gut microbiota modulates immune cell distribution and function by regulating bone marrow myeloid cell differentiation. In the absence of gut microbiota, myeloid cell differentiation is impaired. This results in reduced bacterial clearance and increased susceptibility to allergic diseases, such as asthma (28, 29) Gut microbiota modulates T cell differentiation by regulating dendritic cell (DC) maturation and function. Specifically, gut microbiota induces DCs to express specific co-stimulatory molecules and cytokines, thereby promoting Th1, Th2, and Th17 cell differentiation. In asthma, excessive Th2 cell activation and abnormal Th17 cell function are strongly linked to airway inflammation (29). For the shared pathological mechanisms underlying FI and asthma, supplementation with probiotics or prebiotics may restore intestinal microbial balance, strengthen the intestinal barrier, reduce bacterial translocation and systemic inflammation, and thereby alleviate symptoms of both FI and asthma. Drugs or nutritional supplements with epithelial-repairing properties—such as glutamine and zinc—could exert therapeutic effects on both conditions. Glutamine serves as a critical energy source for intestinal epithelial cells, promoting their repair and regeneration, and enhancing intestinal barrier integrity. Zinc is crucial for synthesizing and activating numerous enzymes and is essential for maintaining epithelial cell structure and function. Zinc supplementation may restore epithelial barrier function in both the airways and intestines, thereby potentially reducing inflammatory responses.

Our study identified age as a significant factor influencing the association between FI and asthma. With advancing age, individuals undergo various neuromuscular changes, including diminished anal resting and squeeze pressures, reduced rectal compliance, impaired rectal sensation, and increased sensory thresholds (30, 31). These physiological changes may increase the susceptibility to FI. The mechanisms underlying the impact of aging on asthma may include a decline in pulmonary elasticity leading to increased airflow limitation (32), reduced response to asthma treatment, and an inflammatory pattern mediated by TH1 and TH17 cells resulting in reduced corticosteroid sensitivity (33). These factors may increase susceptibility to asthma among middle-aged and elderly individuals (34).

There exists a gender disparity in the association between FI and asthma, with women showing a higher likelihood of experiencing the relationship between FI and asthma. Some assumptions suggest that pelvic tissue damage resulting from pregnancy and childbirth may pose a potential risk factor for FI. However, extensive research has not fully explored such associations (35, 36). According to research, vaginal delivery can lead to anal sphincter muscle damage in women (37), particularly during their first vaginal delivery when the risk of anal sphincter rupture is highest. However, with each subsequent delivery, there is an increased likelihood of pelvic nerve injury (37). It has been found that vaginal delivery is not directly associated with FI, whereas the damage to the anal sphincter acts as an independent risk factor for FI (38). Therefore, the role of obstetric injury in the occurrence of FI among women remains a subject of controversy and requires further exploration.

Asthma is more common in adult women, potentially due to differences in physiological structure and sex hormone levels. Compared to men, women have smaller and lighter lungs. Lung structure directly affects ventilatory capacity. This observation remains true even when comparing individuals of the same height. Notably, the capacity of female lungs may be partially attributed to a lower alveolar count (39). Despite having smaller lungs than men, women exhibit higher expiratory flow rates, thus increasing the likelihood of respiratory symptoms (40). Fluctuations in sex hormone levels during the menstrual cycle may play an important role in asthma development. Estrogen and progesterone directly regulate immune pathways involved in asthma pathogenesis, while testosterone may exert protective effects on asthma-related inflammatory processes, although the exact mechanisms are not fully understood (41). During specific phases of the menstrual cycle and pregnancy, some women with asthma may experience exacerbated symptoms. Particularly during the luteal phase, when estrogen and progesterone levels increase, asthma symptoms can periodically intensify, affecting approximately 11%–45% of women (42). These two hormones are capable of directly modulating the immune pathways involved in asthma pathogenesis, thereby heightening airway responsiveness to various stimuli and increasing the risk of asthma exacerbations. For female patients with FI, given that their intestinal and pelvic floor functions may already exhibit certain abnormalities, hormonal fluctuations influencing airway reactivity may further predispose them to the induction or exacerbation of asthma symptoms, thereby reinforcing the link between FI and asthma. A study analyzed the responsiveness of airways to acetylcholine during the follicular and luteal phases, finding that 30% of asthmatic women show significant reactivity to acetylcholine during the follicular phase. This finding supports the notion that female bronchial hyperresponsiveness is associated with hormonal levels (43).

Our study used the nationally representative NHANES database across three research cycles. We incorporated additional covariates to investigate the relationship between FI and asthma risk. This study also presents several limitations. First, the cross-sectional design limits our ability to establish the temporal sequence between FI and asthma. Although we observed a significant association, it remains unclear whether FI precedes asthma onset or vice versa. Second, despite adjusting for multiple confounders, residual confounding from unmeasured variables may influence the observed association. Third, although the potential mechanisms discussed in this study are grounded in theoretical evidence, their interpretation remains partially speculative. Further validation and clarification of these mechanisms will necessitate additional basic research, longitudinal studies, and intervention studies. Finally, some covariates that may affect asthma were not directly included, such as data on pelvic floor disorders, which may have impacted the results.

## 5 Conclusion

Our study has found a significant correlation between FI and asthma, particularly among females, middle-aged and older adults, and individuals with obesity. While this association warrants clinical attention, further longitudinal research is needed to determine whether interventions targeting FI could influence asthma risk. Consequently, it is crucial to closely monitor respiratory symptoms in individuals with FI. Early assessment and management of FI may improve overall health outcomes, but causality remains to be established.

## Data Availability

The original contributions presented in this study are included in this article/Supplementary material, further inquiries can be directed to the corresponding author.

## Footnotes

1
http://www.R-project.org

## References

[1] Khalili R Bartell S Hu X Liu Y Chang H Belanoff C Early-life exposure to Pm(2.5) and risk of acute asthma clinical encounters among children in massachusetts: A case-crossover analysis. *Environ Health.* (2018) 17:20. 10.1186/s12940-018-0361-6 29466982 PMC5822480

[2] Mazurek J Syamlal G. Prevalence of asthma, asthma attacks, and emergency department visits for asthma among working adults - National health interview survey, 2011-2016. *MMWR Morb Mortal Wkly Rep.* (2018) 67:377–86. 10.15585/mmwr.mm6713a1 29621204 PMC5889242

[3] Nurmagambetov T Kuwahara R Garbe P. The economic burden of asthma in the United States, 2008-2013. *Ann Am Thorac Soc.* (2018) 15:348–56. 10.1513/AnnalsATS.201703-259OC 29323930

[4] Zhang W Li W Du J. Association between dietary carotenoid intakes and the risk of asthma in adults: A cross-sectional study of Nhanes, 2007-2012. *BMJ Open* (2022) 12:e052320. 10.1136/bmjopen-2021-052320 35701051 PMC9198789

[5] Rockwood T. Incontinence severity and Qol scales for fecal incontinence. *Gastroenterology.* (2004) 126:S106–13. 10.1053/j.gastro.2003.10.057 14978646

[6] Whitehead W Borrud L Goode P Meikle S Mueller E Tuteja A Fecal incontinence in Us adults: Epidemiology and risk factors. *Gastroenterology.* (2009) 137:512-7–517.e1-2. 10.1053/j.gastro.2009.04.054 19410574 PMC2748224

[7] Ditah I Devaki P Luma H Ditah C Njei B Jaiyeoba C Prevalence, trends, and risk factors for fecal incontinence in United States adults, 2005-2010. *Clin Gastroenterol Hepatol.* (2014) 12:636–43.e1-2. 10.1016/j.cgh.2013.07.020 23906873

[8] Rao S. Current and emerging treatment options for fecal incontinence. *J Clin Gastroenterol.* (2014) 48:752–64. 10.1097/mcg.0000000000000180 25014235 PMC4166012

[9] Xu X Menees S Zochowski M Fenner D. Economic cost of fecal incontinence. *Dis Colon Rectum.* (2012) 55:586–98. 10.1097/DCR.0b013e31823dfd6d 22513438

[10] Black H Mendoza M Murin S. Thoracic manifestations of inflammatory bowel disease. *Chest.* (2007) 131:524–32. 10.1378/chest.06-1074 17296657

[11] Tzanakis N Tsiligianni I Siafakas N. Pulmonary involvement and allergic disorders in inflammatory bowel disease. *World J Gastroenterol.* (2010) 16:299–305. 10.3748/wjg.v16.i3.299 20082474 PMC2807949

[12] von Elm E Altman D Egger M Pocock S Gøtzsche P Vandenbroucke J. The strengthening the reporting of observational studies in epidemiology (Strobe) statement: Guidelines for reporting observational studies. *Lancet.* (2007) 370:1453–7. 10.1016/s0140-6736(07)61602-x 18064739

[13] Huang Y Wu M Wang Y Wei J. The influence of constipation on asthma: A real-world, population-based cohort study. *Int J Clin Pract.* (2021) 75:e14540. 10.1111/ijcp.14540 34132008

[14] Horng S Chou Y Huang N Fang Y Chou P. Fecal incontinence epidemiology and help seeking among older people in Taiwan. *Neurourol Urodyn.* (2014) 33:1153–8. 10.1002/nau.22462 24000147

[15] Gersemann M Wehkamp J Stange E. Innate immune dysfunction in inflammatory bowel disease. *J Intern Med.* (2012) 271:421–8. 10.1111/j.1365-2796.2012.02515.x 22324936

[16] Holgate S. Epithelium dysfunction in asthma. *J Allergy Clin Immunol.* (2007) 120:1233–44. 10.1016/j.jaci.2007.10.025 18073119

[17] Bernstein C Wajda A Blanchard J. The clustering of other chronic inflammatory diseases in inflammatory bowel disease: A population-based study. *Gastroenterology.* (2005) 129:827–36. 10.1053/j.gastro.2005.06.021 16143122

[18] Benezech A Desmazes-Dufeu N Baumstarck K Bouvier M Coltey B Reynaud-Gaubert M Prevalence of Fecal Incontinence in Adults with Cystic Fibrosis. *Dig Dis Sci.* (2018) 63:982–8. 10.1007/s10620-017-4825-2 29086331

[19] Henderson P van Limbergen J Schwarze J Wilson D. Function of the intestinal epithelium and its dysregulation in inflammatory bowel disease. *Inflamm Bowel Dis.* (2011) 17:382–95. 10.1002/ibd.21379 20645321

[20] Straub R Antoniou E Zeuner M Gross V Schölmerich J Andus T. Association of autonomic nervous hyperreflexia and systemic inflammation in patients with Crohn’s disease and ulcerative colitis. *J Neuroimmunol.* (1997) 80:149–57. 10.1016/s0165-5728(97)00150-1 9413271

[21] Valdetaro L Thomasi B Ricciardi M Santos K Coelho-Aguiar J Tavares-Gomes A. Enteric nervous system as a target and source of Sars-Cov-2 and other viral infections. *Am J Physiol Gastrointest Liver Physiol.* (2023) 325:G93–108. 10.1152/ajpgi.00229.2022 37253656 PMC10390051

[22] Round A Joo M Barakso C Fallah N Noonan V Krassioukov A. Neurogenic bowel in acute rehabilitation following spinal cord injury: Impact of laxatives and opioids. *J Clin Med.* (2021) 10:1673. 10.3390/jcm10081673 33919666 PMC8069767

[23] Rao S. Pathophysiology of adult fecal incontinence. *Gastroenterology.* (2004) 126:S14–22. 10.1053/j.gastro.2003.10.013 14978634

[24] Fisher J Vincent S Gomeza J Yamada M Wess J. Loss of vagally mediated bradycardia and bronchoconstriction in mice lacking M2 or M3 muscarinic acetylcholine receptors. *Faseb J.* (2004) 18:711–3. 10.1096/fj.03-0648fje 14977875

[25] Kummer W Fischer A Kurkowski R Heym C. The sensory and sympathetic innervation of guinea-pig lung and trachea as studied by retrograde neuronal tracing and double-labelling immunohistochemistry. *Neuroscience.* (1992) 49:715–37. 10.1016/0306-4522(92)90239-x 1380140

[26] Bradley K Finsterbusch K Schnepf D Crotta S Llorian M Davidson S Microbiota-driven tonic interferon signals in lung stromal cells protect from influenza virus infection. *Cell Rep.* (2019) 28:245–56.e4. 10.1016/j.celrep.2019.05.105 31269444

[27] Mindt B DiGiandomenico A. Microbiome modulation as a novel strategy to treat and prevent respiratory infections. *Antibiotics (Basel).* (2022) 11:474. 10.3390/antibiotics11040474 35453224 PMC9029693

[28] Hill D Siracusa M Abt M Kim B Kobuley D Kubo M Commensal bacteria-derived signals regulate basophil hematopoiesis and allergic inflammation. *Nat Med.* (2012) 18:538–46. 10.1038/nm.2657 22447074 PMC3321082

[29] Li R Li J Zhou X. Lung microbiome: New insights into the pathogenesis of respiratory diseases. *Signal Transduct Target Ther.* (2024) 9:19. 10.1038/s41392-023-01722-y 38228603 PMC10791971

[30] Fox J Fletcher J Zinsmeister A Seide B Riederer S Bharucha A. Effect of aging on anorectal and pelvic floor functions in females. *Dis Colon Rectum.* (2006) 49:1726–35. 10.1007/s10350-006-0657-4 17041752

[31] Ryhammer A Laurberg S Bek K. Age and anorectal sensibility in normal women. *Scand J Gastroenterol.* (1997) 32:278–84. 10.3109/00365529709000207 9085467

[32] Gelb A Yamamoto A Verbeken E Nadel J. Unraveling the pathophysiology of the asthma-copd overlap syndrome: Unsuspected mild centrilobular emphysema is responsible for loss of lung elastic recoil in never smokers with asthma with persistent expiratory airflow limitation. *Chest.* (2015) 148:313–20. 10.1378/chest.14-2483 25950858

[33] Dunn R Busse P Wechsler M. Asthma in the elderly and late-onset adult asthma. *Allergy.* (2018) 73:284–94. 10.1111/all.13258 28722758

[34] Wang J Zhang X Zhang L Liu Y Wang G Zhang H Age-related clinical characteristics, inflammatory features, phenotypes, and treatment response in asthma. *J Allergy Clin Immunol Pract.* (2023) 11:210–9.e3. 10.1016/j.jaip.2022.09.029 36191867

[35] Shin G Toto E Schey R. Pregnancy and postpartum bowel changes: Constipation and fecal incontinence. *Am J Gastroenterol.* (2015) 110:521–9. 10.1038/ajg.2015.76 25803402

[36] Schei B Johannessen H Rydning A Sultan A Mørkved S. Anal incontinence after vaginal delivery or cesarean section. *Acta Obstet Gynecol Scand.* (2019) 98:51–60. 10.1111/aogs.13463 30204238

[37] Donnelly V Fynes M Campbell D Johnson H O’Connell P O’Herlihy C. Obstetric events leading to anal sphincter damage. *Obstet Gynecol.* (1998) 92:955–61. 10.1016/s0029-7844(98)00255-5 9840557

[38] De Leeuw J Vierhout M Struijk P Hop W Wallenburg H. Anal sphincter damage after vaginal delivery: Functional outcome and risk factors for fecal incontinence. *Acta Obstet Gynecol Scand.* (2001) 80: 830–4. 10.1034/j.1600-0412.2001.080009830.x 11531634

[39] Thurlbeck W. Postnatal human lung growth. *Thorax.* (1982) 37:564–71. 10.1136/thx.37.8.564 7179184 PMC459376

[40] Pignataro F Bonini M Forgione A Melandri S Usmani O. Asthma and gender: The female lung. *Pharmacol Res.* (2017) 119:384–90. 10.1016/j.phrs.2017.02.017 28238829

[41] Jenkins C Boulet L Lavoie K Raherison-Semjen C Singh D. Personalized treatment of asthma: The importance of sex and gender differences. *J Allergy Clin Immunol Pract.* (2022) 10:963–71.e3. 10.1016/j.jaip.2022.02.002 35150902

[42] Sánchez-Ramos J Pereira-Vega A Alvarado-Gómez F Maldonado-Pérez J Svanes C Gómez-Real F. Risk factors for premenstrual asthma: A systematic review and meta-analysis. *Expert Rev Respir Med.* (2017) 11:57–72. 10.1080/17476348.2017.1270762 27935742

[43] Troisi R Speizer F Willett W Trichopoulos D Rosner B. Menopause, postmenopausal estrogen preparations, and the risk of adult-onset asthma. A prospective cohort study. *Am J Respir Crit Care Med.* (1995) 152:1183–8. 10.1164/ajrccm.152.4.7551368 7551368

