# Supplementary material for: The association between fecal incontinence and asthma among adult Americans: evidence from NHANES 2005–2010
Source: Front Med (Lausanne). 2025 Jun 3;12:1564308. doi: 10.3389/fmed.2025.1564308 (PMC12170319; doi:10.3389/fmed.2025.1564308)
Supplement: Supplementary file 1 [file Table_1.docx]

Supplementary Material

# Supplementary Tables

| Variable | Without FI  OR (95%CI) | With FI  OR (95%CI) | *P-*value |
| --- | --- | --- | --- |
| Model I | 1(Ref) | 1.87(1.45-2.42) | <0.001 |
| Model II | 1(Ref) | 1.95(1.5-2.55) | <0.001 |
| Model III | 1(Ref) | 1.83(1.4-2.39) | <0.001 |
| Model IV | 1(Ref) | 1.83(1.4-2.4) | <0.001 |
| Model V | 1(Ref) | 1.65(1.25-2.19) | <0.001 |

Model I: no adjusted.

Model II: adjusted for age + gender + race/ethnicity + marital status + education level.

Model III: Model II+ smoking status + alcohol consumption + physical activity + BMI + PIR.

Model IV: Model III + plain water + energy + dietary fiber.

Model V: Model IV + diabetes + hypertension + hyperlipidemia + heart disease + kidney disease + depression + emphysema.
